# Towards personalized integrated dementia care: a qualitative study into the implementation of different models of case management

**DOI:** 10.1186/1471-2318-14-84

**Published:** 2014-07-08

**Authors:** Lisa D Van Mierlo, Franka JM Meiland, Hein PJ Van Hout, Rose-Marie Dröes

**Affiliations:** 1 VU University Medical Centre, Department of General Practice and Elderly Care Medicine, EMGO + Institute for Health and Care Research, Van der Boechorststraat 7, 1081 BT Amsterdam, The Netherlands; 2 VU University Medical Centre, Department of Psychiatry, Alzheimer Centre, EMGO Institute for Health and Care Research, Valeriusplein 9, 1075 BG Amsterdam, The Netherlands

**Keywords:** Case management, Dementia care, Implementation, Process analysis

## Abstract

**Background:**

The aim of this process evaluation was to provide insight into facilitators and barriers to the delivery of community-based personalized dementia care of two different case management models, i.e. the linkage model and the combined intensive case management/joint agency model. These two emerging dementia care models differ considerably in the way they are organized and implemented. Insight into facilitators and barriers in the implementation of different models is needed to create future guidelines for successful implementation of case management in other regions.

**Methods:**

A qualitative case study design was used; semi-structured interviews were conducted with 22 stakeholders on the execution and continuation phases of the implementation process. The stakeholders represented a broad range of perspectives (i.e. project leaders, case managers, health insurers, municipalities).

**Results:**

The independence of the case management organization in the intensive model facilitated the implementation, whereas the presence of multiple competing case management providers in the linkage model impeded the implementation. Most impeding factors were found in the linkage model and were related to the organizational structure of the dementia care network and how partners collaborate with each other in this network.

**Conclusions:**

The results of this process evaluation show that the intensive case management model is easier to implement as case managers in this model tend to be more able to provide quality of care, are less impeded by competitiveness of other care organizations and are more closely connected to the expert team than case managers in the linkage model.

## Background

Over the past years, various forms of community-based case management of dementia have emerged in different regions in the Netherlands. Unlike disease management, case management is especially suitable for managing complex situations that do not fit into a single protocol and are difficult to manage, such as care for people with dementia and informal caregivers [1,2]. Case management is characterized by long-term support and guidance both for community-dwelling people with dementia and their informal caregivers.

Case managers can provide information, support, counseling and coordination of care based on the individual needs of the person with dementia and their informal caregiver. Case management can therefore be described as a type of person-centered care. How this care is delivered and the effects it has depends in part on the way case management is organized. Worldwide, there are different case management models that are implemented in various ways [3,4]. These differences are related to e.g. the type of care case managers provide (e.g. assessment, education, liaising, counseling), degree of collaboration with other care professionals, integration in a multidisciplinary team and professional background of case managers. This heterogeneity may explain the mixed effects of case management in dementia to date [5,6].

In the Netherlands the urgency of further implementation of case management has been emphasized in the recent publication of the 'Dementia Care Standard’ [7]. It describes case management as a standard for good care and support for people with dementia and their caregivers. To implement case management successfully, it is important to know the factors that facilitate or impede this implementation. Minkman *et al.*[8] were the first to study success and failure factors of the implementation of Dutch case management programs. Success factors they described included investment in a strong provider or care network and good personal connections with professionals, expert knowledge of the case managers, and embedding case management in a multidisciplinary team. Failure factors described were: distrust of the program by and competition between local care providers, inadequate funding and little involvement of primary care doctors. However, this study did not distinguish between different types of case management models.

There are two prominent dementia case management models in the Netherlands [9]: the first is the linkage model that consists of a dementia network in which multiple case management providers are active and the case manager acts as a mediator between the client and the multiple care agencies [10]. The second is the combined intensive case management and joint agency model (in short: the intensive model) which can be described as a dementia network where both case management and any additional care services (such as diagnostics and medical treatment) are embedded in one independent organization. In contrast with the linkage model where case management is often introduced after diagnosis, case management in the intensive model may start before the diagnosis.

In this article we will describe differences and similarities between these two prominent case management models with regard to facilitators and barriers to the implementation of case management. This study is important because while case management is growing exponentially, there are still no general guidelines for successful implementation. Insight into the facilitators and barriers of the implementation of two different case management models will help to decide which model is more effective, and will contribute to the development of guidelines for the implementation of case management. This will help other regions to implement case management successfully [11-13].

We will compare the results of our study with results from a recent study by Nivel [14] in which different types of case management in thirteen regions of the Netherlands were explored. The Nivel report describes success factors, points of improvement for case management, as well as essential factors for implementation, possible solutions to realize these factors and their advantages and disadvantages. However, the Nivel study did not describe facilitating and impeding factors for the implementation of case management in different models, nor did it investigate different phases of implementation.

The process evaluation described in this article is based on the theoretical model of adaptive implementation [11,15,16]. This model describes external factors (e.g. characteristics of the intervention, operational preconditions, personal and financial resources) that can influence the implementation of case management during various phases (preparation, execution and continuation), and it differentiates between influencing factors on different levels in each of these phases: micro level (care provider, person with dementia and informal carer), meso level (collaboration between care providers/organizations) and macro level (legal and financial framework).

The research questions in this study are: which factors facilitate and impede the implementation of two different case management models? Do the two models differ with regard to the identified facilitators and barriers of implementation? A further question was which of the models best enables case managers to provide personalized care?

## Method

### Design

We used a qualitative multiple case study design [17] that included semi-structured interviews with stakeholders who represented all stakeholder perspectives (i.e. project leaders, case managers, insurance companies, municipalities, patient & caregiver advocacy organizations) in various phases of the implementation process. The Medical Ethics Committee of the VU University Medical Center approved the study protocol.

### Setting

The study was conducted in the period of July 2010 to April 2012 in seven regions in the Netherlands that applied one of the two case management models under study.

The most important factor that determined which case management model is used in a region is the presence of one or multiple care organizations that could perform case management. Regions with one major care organization could integrate case management within that single organization (intensive case management model). Regions with multiple care organizations that all wanted to perform case management implemented the linkage model in which many case management providers are active.

In Amsterdam Nieuw-West, Amsterdam Zuid-Oost, Amstelveen and Flevoland Oost, the linkage model was executed and studied. In Noord Holland Noord, Haarlem and Almere, the intensive model was executed and studied.

Table 1 provides the main characteristics of both case management models under study.

**Table 1:** Comparison of the two main case management models in the COMPAS* study

**Characteristics of different models**	**Linkage model**	**Intensive case management/joint agency model**
**Central point for registration of cognitively impaired persons**	New clients are referred by GP or medical specialist to the central registration point after diagnosis	New clients are referred by GP or medical specialist to the Multidisciplinary team at central registration point before or after diagnosis
**Possibility to diagnose dementia**	No, CM generally starts after diagnosis	By Multidisciplinary team
**Starting point of case management**	After diagnosis	Also possible before diagnosis; e.g. in case of MCI or suspicion of dementia.
**Delivery of services**	Independent and competitive organizations that often differ regarding case manager tasks and type of employment.	Mainly by one organization that provides uniform case manager tasks
**Multidisciplinary team**	Intramural or extramural expert team that case managers can consult. Not always operating in the same organization. Frequency of consultation varies	Elderly care physicians, neuropsychologist, neurologist, geriatrician, psychiatrist, dementia consultant all work within the same organization as case managers
**Financing**	Annual contracts with insurance companies. Funding is provided based on the “Law on Exceptional Medical Expenses” (AWBZ) as well as municipalities (WMO).	Annual contracts with insurance companies. Funding is provided based on the Law on Exceptional Medical Expenses (AWBZ) as well as municipalities (WMO). Sometimes diagnostics and treatment tasks are funded by the Health Insurance Act (Zvw) and certain case manager tasks are covered by the Diagnostic Treatment Combinations (DBC).

(CM = case management, GP = general practitioner, WMO = Social Support Act).

(*The COMPAS study investigates clinical, cost-effectiveness and process outcomes between the case management models and usual care).

### Data collection

**Figure 1:**
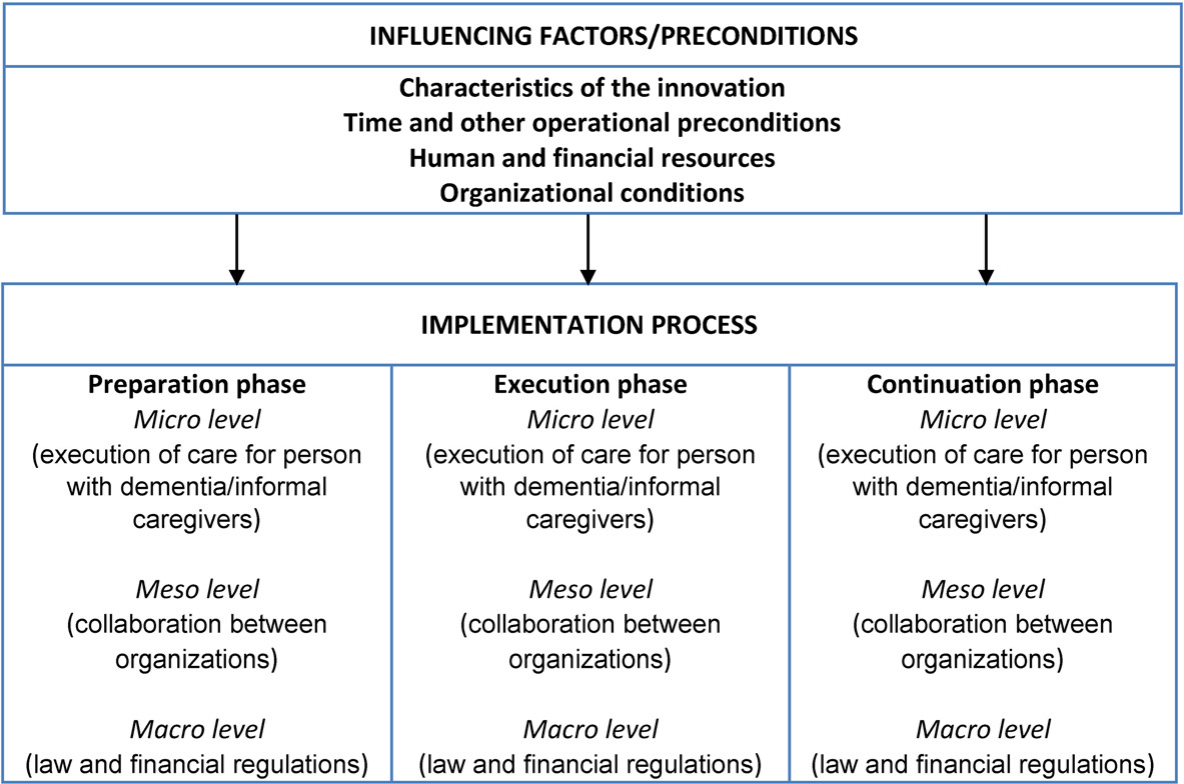
**Theoretical model of adaptive implementation.** (Droës et al., 2003 [15]; Meiland et al., 2004 [11]).

Semi-structured interviews were conducted, with questions derived from the theoretical model of Adaptive Implementation (see Figure 1). Prior to each interview the most relevant questions for each key figure were selected based on their area of expertise and involvement in the implementation process.

In this article we describe the traced facilitators and barriers of the preparation and execution phases combined. As case management was implemented several years ago in the included regions, it was difficult to make a reliable distinction between the two phases.

The stakeholders were selected via 'purposive sampling’ [18], to promote *qualitative rigor*. Stakeholders were representatives from different organizations involved in case management, or the dementia collaboration network surrounding case management. The aim was to interview at least one type of key figure representing two different models per region. Prior to their participation in the study oral informed consent was acquired from all participants. A total of 22 stakeholders representing 8 stakeholder perspectives were interviewed: participants 1 and 2 were case managers, participants 3 through 7 were project leaders and care coordinators of care organizations, participants 8 and 9 were general practitioners, participants 10 and 11 were stakeholders from health insurance companies, participants 12 and 13 were stakeholders from the mental health service, participants 14 and 15 were program coordinators of a day care center, participants 16,17 and 18 were stakeholders from regional departments of Alzheimer Netherlands, participants 19, 20 and 21 were stakeholders from municipalities and participant 22 was a representative from an informal caregiver support organization (representing the linkage model).

### Analysis

On average, the interviews lasted 60 to 80 minutes. They were transcribed ad verbatim and entered into the qualitative computer program Atlas-ti [19]. The directed content analysis [20] was used as the methodological framework for exploration of the data. All interviews were coded independently by two assessors who assigned key words to extracts from the text. These key words were part of a detailed checklist that was constructed based on the theoretical adaptive implementation model and contained subjects related to possible facilitating or impeding factors. The coding of the first interviews was discussed in detail, which resulted in sharpening of the interview questions and the key words. Additional keywords were also added. Subsequently all codes assigned by the assessors were incorporated in Atlas-ti. Next, all extracts of key words were inventoried and analyzed. The results were summarized per case management model and reflected facilitating and impeding factors for the implementation of the two case management models for each level of implementation (micro, meso, macro) and the different phases of the implementation process (preparation, execution and continuation). These results were discussed in the project group.

To compare our results with the results of the national case management study of Nivel, information on facilitators and barriers to the implementation of case management was extracted from the Nivel report [14].

Our study adheres to the RATS guidelines on reporting qualitative research. Qualitative rigor was ensured by a number of steps. We created an audit trail of coding and analysis decisions, including evolving coding schemes. Credibility of the research was promoted by peer debriefing among colleagues of the research department [21]. We ensured reliability and validity by including 7 distinct regions across the Netherlands and using three different investigators to analyze the data. Member checks were performed with each stakeholder after an interview was conducted. This means interview data was checked with stakeholders for accuracy after the interview. Furthermore, each interview was double coded by two independent investigators from a group of three different investigators.

## Results

### Comparing the linkage and intensive model

Tables 2 and 3 show the main differences in facilitators and barriers between the two case management models as inventoried in this process evaluation, compared with each other as well as with the Nivel results. When necessary, these differences are explained further in the text. Table 2 shows external factors and preconditions that can influence the implementation during the whole implementation process. Table 3 shows factors that influence the implementation during the combined preparation and execution phase and the continuation phase. All factors in Table 3 were synthesized into the following themes: case manager characteristics, content of case management, organizational structure, collaboration with dementia care partners, quality of care, and law & legislation and financing. In addition, we also describe factors that influenced the implementation in both models. As the Nivel study did not focus specifically on different phases of implementation, they did not report any factors related to the continuation phase.

**Table 2:** External factors and preconditions before starting the implementation that can influence the implementation process

		**Intensive**	**Linkage**	**Nivel**
**Characteristics of case management**				
*Facilitating*	Using existing non-dementia case management models as example	+		
*Impeding*	Disagreement about content of case manager tasks		-	-
	Partners do not see the added value of a case manager who only mediates		-	
	Speed of implementation depends on mentality and cultural values of the region		-	
**Time and other operational preconditions**				
*Facilitating*	Sufficient time to set up an organizational structure		+	+
*Impeding*	Professionals don't have innovation time; consensus among many collaboration partners takes time	-		
	No clear guidelines for implementation		-	-
**Human and financial resources**				
*Facilitating*	Retraining district nurses to become case managers facilitates collaboration with the GP as they have pre-existing partnerships	+		
	Presence of a clear initiator of the implementation	+		
*Impeding*	Proliferation of different types of case managers created friction among providers		-	-
	Lack of clarity about the role of the project leader (not knowing who is their superior)		-	
**Organizational conditions**				
*Facilitating*	Embedding case management in Mental Health Care promotes collaboration	*+*		
	Embedding the multidisciplinary expert team in case management organization	+		
	Good collaboration between case managers from competitive providers provides the opportunity to learn from each other		+	+
	Case managers from one provider all working in the same room enhances sparring and consultation		+	
	Presence of a Board of Representatives to guide the dementia care network.		+	+
	Presence of fixed stakeholders at partners in the dementia care network whom case managers can contact	+		+
*Impeding*	Presence of competitive providers of case management within the dementia care network		-	-
	Different interests of the Board of Representatives; incomplete attendance during meetings; members without mandate to make decisions.		-	
	Expert team doe not function properly; difficult to reach clinicians as members participate only a few hours per week.	-		
	Lack of clarity about who is responsible for what aspects of implementation and collaboration		-	
	Only incorporating dementia care partners with the strongest pre-existing relationships at the start	-		

+ = facilitating factor, - = impeding factor, a blank cell means a factor was not extracted from interviews in regions within this model or the Nivel study.

**Table 3:** Facilitating and impeding factors during the execution and continuation phase

			***Intensive***		***Linkage***		**Nivel**
**Case manager characteristics**			**Execution**	**Continuation**	**Execution**	**Continuation**	
**Micro level**	*Facilitating*	Large case manager team made it possible to consider individual competences and a differentiated offer of tasks		+			
		Increase in experience enabled case managers to discuss clients without the expert team				+	
	*Impeding*	Case manager with a dual role encounter time restraints and run a burn out risk			-		
		Creating additional tasks + increase of caseloads leads to higher work pressure (especially when case managers have dual-jobs). No clear agreements about who is responsible for additional tasks				-	
		Individual differences + increase in case manager team makes uniform way of practice difficult		-			
		Difficulty hiring case managers with the right qualifications for the job		-			
		Increase in case managers means less time per case manager to discuss clients in expert team				-	
**Content of case management**							
**Micro level**	*Facilitating*	Protocols that allow case managers to indicate which clients have a priority for nursing home admission		+			
	*Impeding*	Difficulty to approach expert team when imbedded in an intramural setting and/or when crossing over to a different organization			-		
		Health care agencies provide funding for a fixed number of clients but caseloads of case managers often exceed that number				-	
		Indistinct quality demands on case manager tasks			-	-	
		No agreement on the content of the care plan and no uniform registration system				-	
**Organizational structure**							
**Meso level**	*Facilitating*	Guarding and continuing the integration of case management. Preconditions are: well profiled case management, good collaboration between partners and overall satisfaction of case management by partners		+			+
		A platform of directors of dementia network partners who can develop new initiatives in case management		+			
		Creating a production plan for municipalities to provide insight into what type of care they purchase		+			
	*Impeding*	No clear referral procedures			-		
		Not documenting what happens to responsibilities for organizational tasks on a structural basis				-	
		A change in the board or employees in dementia network partners can change their motivation		-			
**Collaboration with dementia care partners**							
**Meso level**	*Facilitating*	Transparency about case management practice towards dementia partners	+	+			
		Regular meetings with social psychiatric nurses from Mental Health Care to discuss and solve collaboration issues	+				
		Exchanging knowledge between case managers and other disciplines increases cohesion				+	
		Using existing collaboration networks to build on, e.g. networks between general practitioners and district nurses			+		
		Collaboration with general practitioners, home care and day care centers can be strengthened by being each other's eyes and ears		+			
	*Impeding*	Partners have difficulty seeing case managers as equivalent to social psychiatric nurses, with whom they have experience			-		
		No collaboration between the municipalities and the health care agency		-			-
		Lack of transparency about division of funding from health care agencies to case management providers				-	
**Quality of care**							
**Meso level**	*Facilitating*	Staying focused on individual needs of clients when discussing with care partners	+				+
		Commitment of the Alzheimer's Association + delegation of patients and case managers		+			+
	*Impeding*	Influence of the government who advocates primary care and care that is not disease specific				-	
		As social psychiatric nurses hand over clients and tasks to case managers they lose touch with psychogeriatrics and the social chart even though clients would benefit from good collaboration between case managers and social psychiatric nurses				-	
		Referral by case managers based on competing interests of providers instead on what clients need.				-	-
**Law & legislation/Financing**							
**Meso level**	*Impeding*	Regions made up the balance too late for an effective transfer of funds from regions with an excess of funds				-	
		Financial agreement that case management can only start after diagnosis			-		
		Smaller municipalities can easily drop their funding when the pressure rises, creating a gap		-			
		Without project funding administrational support for case managers was dropped				-	
**Macro level**	*Facilitating*	Pilot funding gave regions space to develop case management (but also caused a diversity in practice)			+		
		Part of the financing from Mental Health Care could be adopted for case management	+				
		Introduction of the DBC: it included tasks that case managers perform		+			+
		Redistributing funding across regions by health care agency based on needs of regions				+	
	*Impeding*	As project funding ended, project leaders and coordination points were omitted				-	
		Lack of full insurance cover for case management led to fragmentation of financial support		-			
		In some regions diagnostics and treatment are funded by the Health Insurance Act, but not in all of them		-			
		DBC does not cover all case management tasks		-			

+ = facilitating factor, - = impeding factor, a blank cell means a factor was not extracted from interviews in regions within this model or the Nivel study.

### Influencing factors and preconditions

#### Characteristics of case management

In both models, facilitating factors for implementation were: involving people with dementia and caregivers during the initial preparation phase; investigating their existing care needs in order to ensure case managers can provide tailored dementia care; and a clear added value of case management for informal caregivers and people with dementia compared to care as usual.

#### Time and other operational preconditions

Facilitating factors mentioned in both models were: clear agreement about which organizations or stakeholders are responsible for which organizational tasks before case management is implemented; sufficient time to set up a clear organizational structure (including a coordination contact point, an expert team and case managers); a clear protocol that describes collaboration on the level of primary care; and clear procedures for referral to case management.

#### Human and financial resources

Case manager characteristics that were traced in both models that contributed to the quality of care throughout the implementation are: having dementia experience; enthusiasm for the job; determination; the capacity to show empathy towards the client/informal caregiver; working as a case manager at least 16 hours per week; a certain level of education; participation in an acknowledged training program for case managers. In addition, full-time case managers have more dementia expertise, given their continuous education and substantial caseload, whereas the part-time availability of case managers made them less accessible to informal caregivers or professionals. Finally, care innovation funds provided by the health care agency facilitated the implementation of case management in both models.

Disagreement about which type of employment was desirable (case managers who work full time or part-time but with no additional jobs, or care professionals who work as case managers but also have additional jobs, such as district or practice nurse) impeded the implementation in the linkage model, and the proliferation of different types of case managers created friction between the multiple providers. In the words of two stakeholders:

“We started with six case managers from three different care providers. Then the elderly care advisors from a welfare organization also wanted to be case managers, and there also was a request from two general practices to imbed two practice nurses as case managers. There was a lot of conflict about who was going to do what… (participant 4)”. “Every organization wanted to provide case managers… and everybody thought they could do the best job (participant 3).”

#### Organizational conditions

Essential facilitating factors for the implementation of both case management models are: support and commitment from important partners in the dementia care network (such as municipalities, insurers, general practitioners and day care centers) and creating strong collaborative relationships with them; a clear vision on what the network wants to achieve; keeping all partners in the dementia care network involved throughout the implementation process; and convincing all partners of the value of case management for their own organization as this can contribute to a long-term collaboration within the dementia care network. Furthermore, participation of the general practitioner and having a clear idea of his role in the dementia care network, as well as assigning case managers to specific sub-regions were reported to facilitate collaboration with the general practitioners and to provide case managers with a better overview of community resources.

In the linkage model, guiding and directing the dementia care network by a Board of Representatives proved to be difficult, and it impeded implementation. As one stake holder illustrated:

*“On a management level there was sometimes more competition than collaboration. That did impede things. How do you reach agreement on decisions? What information do you share? How much do you trust each other? And they all have a vision, different visions about the way in which case management should be practiced* (participant 16).”

Furthermore, the presence of multiple care providers is an impeding factor to providing personalized care, as competition between the organizations can make case managers feel pressured to refer clients to the organization they work for even when this is not in the best interest of the client.

### Facilitating and impeding factors in the execution and continuation phase

#### Micro level (case manager characteristics & content of case management)

Facilitating factors in the *execution phase* in both models were: development of expertise by case managers; taking part in an acknowledged case manager education program; support by an expert team; regular and frequent meetings with the case manager team; and enthusiasm and commitment of case managers. Unanimously reported as impeding factors during the *execution phase* were: insufficient knowledge of the value of case management or resistance from people with dementia and informal caregivers; not knowing how to access case management as an informal caregiver. Providing sufficient information on the content of case management was an important facilitating factor in both models, as it improved awareness as well as acceptance of case management.

Case managers who had a dual role in the linkage model (for instance case manager and district nurse) often encountered time constraints. As one participant stated:

*“..because the combination is often too tough, they start to encounter problems. It leads to stress and burn-out* (participant 7)*.”*

Facilitating factors in the *continuation phase* in both models were: the increase in experience and expertise of case managers and an improved knowledge of the social chart. As a result they are better equipped to provide personalized care for people with dementia and their caregivers and for an expansion of their tasks and responsibilities. This in turn leads to improved acceptance and acknowledgement of case managers by the partners in the dementia care network.

Stakeholders in both models acknowledge the need for continuing education or training for case managers. The absence of structural follow-up of education makes this difficult.

Stakeholders in the linkage model reported a lack of agreement on the content of the care plan, and the absence of a uniform registration system for all care providers. As one case manager stated:

*“We try to keep up with the care plans but I always think, who do I write this for? Because only people in our own organization have access to them. Everybody writes their own care plans, they are not integrated. And when I am very busy, keeping the care plan up to date is the first thing I put aside* (participant 2)*.”*

Lastly, stakeholders in the intensive model stated that introducing protocols in collaboration with nursing homes that allow case managers to indicate which clients are in urgent need of admission, and giving those clients priority, led to more control over admissions by case managers and faster placement of clients in need.

#### Meso level (collaboration with dementia care partners, organizational structure and quality of care)

A facilitating factor in the *execution phase* in both models was the distribution of information about the value and practice of case management to dementia network partners. Although only one organization provides case management in the intensive model, there were still competing care providers (such as home care or volunteer organizations).

A crucial strategy to circumvent these interests and facilitate collaboration when discussing clients with other care providers was explained by one stakeholder as follows:

*“You should always turn the conversation back to what a client needs. What can we come up with together to solve his problem? That always helped. Because as soon as we started talking about interests and organizations we would get stuck* (participant 16)*.”*

Impeding factors in the *execution phase* in both models were: collaboration problems with Mental Health Care (with the exception of one region in the intensive model) due to the lack of a clear division of responsibilities between case managers and social psychiatric nurses, and the difficulty social psychiatric nurses had to hand over clients from their caseload to case managers.

Impeding factors in the *continuation phase* in both models were: problems with structural financing after the care innovation funds were withdrawn; no clear agreements about the amount of non-direct client contact compared to direct, reimbursable client contact; and complicated registration systems for case managers.

A facilitating factor in both models and *both implementation phases* was good collaboration between partners in the dementia care network. During the continuation phase, the continued development of case management and collaboration with care partners remained important, as did guarding and continuing the integration of case management in the dementia care network.

Impeding factors in both models and *both implementation phases* were difficulty collaborating with general practitioners and a lack of commitment from municipalities, which impeded welfare organizations to collaborate with case management.

Stakeholders from the intensive model mentioned that during the continuation phase of case management, the collaboration with home care, day care centers and general practitioners could be strengthened by frequent consultation and exchanging knowledge, by providing feedback on changes in the situation of clients. As one participant stated:

*“A case manager needs home care workers who are their eyes and ears, who keep an eye on the situation of the client* (participant 13)*.”*

This builds a strong network around clients and caregivers, but it can also promote early detection of dementia by general practitioners or home care employees who can send a signal to case managers.

In both models continuous investment in communication with general practitioners was a facilitating factor in the *continuation phase*, especially since GPs are become more elderly-minded and are currently more inclined to do case manager tasks themselves or in collaboration with their practice nurse. Together with the influence of current government policy that promotes that care should be more directed towards primary care this means that case managers have to reconsider how they compare and relate to GPs and practice nurses. The fact that the content of case management in the linkage model differs depending on the provider, plus the fact that there is no accepted definition of what case management comprises or who should act as case manager, puts pressure on the concept of case management.

In the intensive model continued transparency of case management practice to dementia care partners was reported to enable those partners to detect existing gaps in the care offer and to facilitate the development of case management and collaboration with them. In the words of a stakeholder:

*“We are very transparent with the information we have. We keep track of our caseloads. This means at the end of each year we can generate figures about how many clients we have, where they come from, how long they have been in our care and where they get additional care. And this is information the dementia care network can use to investigate whether there are gaps in the care network. In turn this may lead to new developments or initiatives* (participant 6).”

#### Macro level (law & legislation and financing)

Facilitating factors in the *execution phase* for both models were care innovation funds provided by the health care agencies; the policy plan of government and health insurers that advocates integrated dementia care; and the National Dementia Program (NDP).

An impeding factor in the *continuation phase* for both models was having problems with structural financing after project funding stopped. The absence of a distinct financing title for case management made it difficult to find a suitable structural solution and led to fragmentation. As the AWBZ (Law on Exceptional Medical Expenses) funding for case management became tighter, and the municipalities covered a substantial part of the funding through the WMO (the Dutch Social Support Act), it became more difficult for regions to expand case management within the dementia care network, and it also threatened the existing case management structure. While large municipalities can be active partners, it is generally more difficult to convince smaller municipalities of the added value of case management, which influences the amount of funding they are willing to provide.

## Discussion

### New contribution of this study

This study provides insight into impeding and facilitating factors that were encountered during the different phases of implementation of two emergent case management models in the Netherlands. The most important influencing factors found were related to the organizational structure and collaboration. The results of this study contribute to knowledge about how the successful implementation of these models differs.

### Organizational structure and collaboration

Overall, the organizational structure in the *intensive model* facilitated the implementation to a higher degree. Structured management by a clear initiator of the implementation could power and develop the dementia network. Collaboration with other care providers in the dementia care network can be optimized in the intensive model by providing transparency towards other care providers about the content of case management and reaching clear agreements about who is responsible for which aspects of client care within the dementia care network. The independence of the case management provider in the intensive model enables case managers to attune care to what is in the best interest of the client instead of what is best for the case management organization.

The organizational structure in the *linkage model* on the other hand, is marked by the presence of multiple case management organizations and other care providers that are directed by a Board of Representatives. Competing interests of the multiple care organizations as well as insufficient decisiveness on the part of the Board makes it difficult to optimize collaboration and facilitate implementation of case management in the linkage model.

### Content of case management

Although the intended roles of case managers are similar in both models (providing guidance, care and referring clients to care services), the content of the provided case management does differ between the models. While the intensive model is based on a comprehensive concept of case management, in line with the national 'Dementia Care Standard’ [4], the linkage model does not meet this standard. Case managers in the linkage model perform fewer tasks than case managers in the intensive model. This is a result of an ongoing disagreement about whether case management should be a person’s only function (intensive model) or can be carried out in a (part-time) dual-employment (linkage model). Case managers with a dual role experience more time constraints, making it difficult to provide the full range of tasks as described in the Dementia Care Standard.

Furthermore, in the intensive model case management is often introduced before diagnosis, which seems to be more beneficial as case managers are able to direct care towards preventing crises, while case managers in the linkage model have to work more reactively and try to solve problems instead of preventing them. Some care provider organizations in the linkage model aimed at providing case management before diagnosis, but they were impeded by funding issues as well as problems tracing people with early symptoms who need a case manager.

### Personalized dementia care

Our results indicate that case managers in the intensive model are better able to provide personalized care for their clients than case managers in the linkage model. This can be explained by the presence of quality of care factors that were mostly found in the intensive model. While some of these factors were present in both models, e.g. investigating existing needs of clients, and personality traits of case managers like determination and being able show empathy towards clients, other factors were only present in one model: full-time case managers in the intensive model, for example, had more dementia expertise to offer than a dual-role case manager in the linkage model. Case managers in the linkage model found it difficult to stay focused on the individual needs of clients, as they can feel pressured to refer clients to their own organization. This may not always be in the best interest of the client. Case managers in the linkage model sometimes had little or no access to an expert team. All these factors are thought to lead to insufficient quality of care. Finally, the absence of - or limited collaboration with - the social psychiatric nurse in the linkage model impaired the quality of care for complex psychiatric clients who would benefit from combined support.

### Financing

In retrospect, one of the main reasons why case management has grown exponentially in many regions in the Netherlands is that project funding was provided to implement case management with enough room to adapt for regional differences. However, this resulted in considerable variation in practice, both between regions and within regions. This lack of uniformity now impedes structural financing for case management, as there is no accepted model of practice. This makes it nearly impossible for case managers to unite and compel Health care agencies or Health care insurers to set up a distinct financing title for case management and to promote the continuation of case management.

### Overview

Overall, stakeholders in the intensive model were more inclined to talk about facilitating factors, whereas stakeholders in the linkage model focused more on impeding factors. Stakeholders from the linkage model often referred to current problems that developed during the implementation, while stakeholders in the intensive model focused more on how well case management was developed and how to develop it further. This suggests that case managers in the linkage model encountered more barriers when trying to provide care than case managers in the intensive model. Based on the results we can conclude that both case management models encounter similar as well as different facilitating and impeding factors, most of which are strongly related to organizational structures and the collaboration between partners in the dementia network. While the implementation in the intensive model is facilitated by the organizational structure and collaboration, this is not the case in the linkage model, where implementation is impeded by the way the dementia care network is organized and collaborates. The presence of multiple case management organizations and care provider organizations with competing interests appeared to be the most significant barrier in the linkage model and it accounts for the majority of impeding factors that were found. The results of the process evaluation suggest that case managers in the intensive model are better able to provide optimal, personalized care for people with dementia and their caregivers than case managers in the linkage model.

### Results in comparison with literature

The results of the process analysis were compared to the results of a large national evaluation study on case management conducted by Nivel [14]. The majority of results that the Nivel study reported on were retraced in one or both models, but our process evaluation also found additional influencing factors. This article focused primarily on important differences between the two models that influenced the implementation of case management.

The problems that were traced often depended on model differences across regions. These findings are in contrast with the Nivel study that stated that neither success factors nor points of improvement were dependent on the type of case management model. Moreover, while the Nivel study explored preconditions for implementation such as 'good collaboration between partners’ or 'an independent role of the case manager’, they did not investigate if these preconditions differed between case management models, nor did they study different phases of implementation.

Our results are largely in line with what was found in the process evaluation on case management by Minkman *et al.*[8] and in the study on essential components of case management by Verkade *et al.*[22]. The success factors described by Minkman *et al.*[8], i.e. a strong provider network, good personal connection with professionals, expert knowledge of case managers and access to an expert team, all proved more difficult to accomplish in the linkage model. The failure factors described by Minkman were present in both models: inadequate or no structural funding and little or no involvement of general practitioners. The described failure factors 'competition for delivering care’ and 'not including patients without a confirmed dementia diagnosis’ were much stronger impeding factors in the linkage model. The preconditions for good quality case management described in Verkade *et al.*[22] were confirmed in both models to be important facilitating factors, although the extent to which they facilitated the quality of care differed between the models. Having a uniform vision on case management, the use of structured methodology by case managers, integrating case management into the dementia care network and firm agreements about shared responsibilities with care partners proved much more difficult in the linkage model. We found no international studies describing facilitating and impeding factors for case management implementation.

A subsequent question of this process evaluation was which model best enables case managers to provide personalized care based on the care needs of clients? Somme *et al.*[3] recently published a review article in which they compared six RCT studies on case management programs to determine their clinical outcomes as well as the case management intensity by using Pacala's Scale [23]. This scale measures 18 predefined functions of case management programs such as “having a caseload below 60”, “case manager or expert team performs initial assessment” or “case manager talks to the primary care physician personally”. Higher scores indicate a higher intensity program. Somme *et al.*[3] concluded that from those six RCTs, two programs were of high intensity. These were also the programs that showed the largest clinical effects (compliance with case manager recommendations and quality of life of people with dementia and informal caregivers), while the low intensity programs showed slight to no effects. The COMPAS results presented here argue in favor of implementation of the intensive case management model. In our study, results on the comparison of clinical outcome measures are not available yet.

The data collection and analysis were based on the theoretical model of implementation [11,15] that describes various phases of implementation on the micro, meso and macro levels as well as external factors that influence implementation. This theoretical model was particularly useful for the process analysis because it takes into consideration that a successful implementation process often depends on regional characteristics, and adaptation to specific situations is often necessary [24,25]. As dementia care networks in the Netherlands differ per region and case management was implemented in different ways, the theoretical model seems a justified method to compare facilitators and barriers to these different implementation processes.

### Limitations of the study

Several limitations have to be considered when interpreting the results. Even though the results indicate a preference for implementing case management based on the intensive model, regional differences can still make it difficult to implement the intensive model. For instance, the variety of regional structures and the presence of market forces can make it difficult to achieve the desired independence in a case management organization.

Another limitation is that although this study describes differences between models, it was not always clear if these differences were actual differences across models. It is also possible that specific factors were present, but were not mentioned by the stakeholders. Furthermore, in this process evaluation we only interviewed stakeholders in regions that had implemented case management. The design was partly retrospective because in most regions the initial execution phase had taken place several years ago. This sometimes made it difficult for stakeholders to think of facilitators and barriers during the beginning of implementation. In this study we looked at facilitating and impeding factors in the implementation of two case management models from the perspectives of care professionals. Interviewing patients and informal caregivers who received case management from the two models might have provided us with different information about the implementation of the models.

Finally, we are as yet unable to indicate whether there is a relationship between the results of the process evaluation and the (cost-)effectiveness of the case management models. However, this will be investigated at a later stage of the COMPAS project.

### Scientific and clinical relevance

This information can help raise awareness in case management organizations and other dementia care network partners about factors that positively or negatively influence the implementation of case management as a means of providing personalized dementia care.

The relevance for psychogeriatric care is that case management organizations that are currently implementing case management (or regions that want to start implementing case management) can benefit from our results.

One of the strengths of this study is that it identifies factors relevant to different phases of implementation. This may help care organizations define adequate implementation strategies during the preparation and execution phase but can also help to ensure long-term continuation of case management.

As distinctive important factors were identified in each phase, such as developing clear referral procedures to case management in the initial execution phase and keeping the expertise of case managers up to date in the continuation phase, project leaders are informed on the key factors that facilitate the implementation in each phase and thus need special attention. Policy recommendations are needed to stimulate and guide the development of case management initiatives. To ensure successful implementation of case management in different regions, the government should promote a uniform model in which case management is provided by an independent organization with a close connection to an expert team (micro level), and where case managers work in close collaboration with other care professionals to stimulate integrated dementia care (meso level). Financial contracts between health insurers and case management providers should be stimulated to obtain full insurance coverage for case managers and eliminate fragmentation of finances (macro level).

Furthermore, collaboration in the dementia care network should be optimized by providing education about case management for partners and clients, and by forming teams of care professionals that focus on providing optimal care for clients instead of focusing on profit for their own organization.

The results of this implementation study with respect to case management models in dementia care may also be of relevance for other health care systems that provide multidisciplinary care (such as chronic disease care or geriatric psychiatry) and where case managers contribute to the effectiveness and efficiency of the provided care.

Although this process evaluation provides new insights into which factors influence the implementation of the delivery of personalized care for people with dementia and informal caregivers, it also points out that regional differences can be the cause of problems during the implementation. However, being aware of these problems in advance allows regions to prepare and intervene at an early stage to prevent them from becoming insoluble.

The increasing numbers of community-dwelling people with dementia emphasize the importance of continuing research on the implementation of effective and efficient personalized care, and strengthening the integrated dementia care network. The results of this process evaluation contribute to this research.

## Conclusions

The results suggest that the implementation of the intensive case management model is preferable to the linkage model as case managers in the intensive model tend to be better able to provide personalized care as well as overall quality of care, are less impeded by competitiveness of other care organizations and are more closely connected to the expert team. However, regional differences and the organization of dementia care networks do not always clear the way for the implementation of the intensive model. The results of this process evaluation do provide renewed insight into facilitating and impeding factors for implementation of two different case management models: the linkage and the intensive model.

## Competing interests

The authors declare that they have no competing interests.

## Authors’ contributions

LVM, FM, HVH and RMD contributed to the development, conceptualization and the design of the study. LVM was responsible for data collection (conducting interviews), transcription, analysis and preparation of the manuscript. FM, HVH and RMD supervised the research process data and provided assistance with data analysis and editing the final manuscript. All authors contributed to the interpretation of the results, and all authors reviewed and approved the final manuscript.

## Pre-publication history

The pre-publication history for this paper can be accessed here:

http://www.biomedcentral.com/1471-2318/14/84/prepub
